# Updated Overview of the Geographical Prevalence of Polycystic Ovary Syndrome by Diagnostic Criteria, Phenotypes, Race, and Ethnicity

**DOI:** 10.3390/ijerph23081085

**Published:** 2026-08-20

**Authors:** Makenna J. O. Noland, Melissa D. Olfert

**Affiliations:** School of Agriculture and Food Systems, Division of Land Grant Engagement, Davis College of Agriculture and Natural Resources, West Virginia University, Morgantown, WV 26506, USA; mo00039@mix.wvu.edu

**Keywords:** polycystic ovary syndrome, prevalence, geographical, phenotypes, diagnostic tools

## Abstract

**Highlights:**

**Public health relevance—How does this work relate to a public health issue?**

- Polyendocrine Metabolic Ovarian Syndrome (PMOS), formerly Polycystic Ovary Syndrome (PCOS), is one of the most common endocrine and metabolic disorders affecting reproductive-aged women worldwide, and is associated with adverse metabolic, reproductive, and psychological health outcomes.
- Through understanding regional and ethnic differences in PCOS, researchers, policymakers, and healthcare providers can identify populations at increased risk and address disparities in diagnosis, healthcare access, and knowledge surrounding PCOS.

**Public health significance—Why is this work of significance to public health?**

- This review is significant to public health because it demonstrates substantial global variation in PCOS prevalence, with estimates varying by geographical location, race/ethnicity, phenotype distribution, and diagnostic criteria used.
- The continued use of inconsistent diagnostic tools limits comparability across studies and may contribute to inaccurate prevalence estimates worldwide, despite both the 2018 and 2023 International Evidence-Based Guidelines for the Assessment and Management of PCOS recognizing the 2003 Rotterdam criteria as the gold standard.

**Public health implications—What are the key implications or messages for practitioners, policy makers, and/or researchers in public health?**

- Greater adoption of the 2003 Rotterdam criteria and increased awareness of international evidence-based guidelines may be needed to improve consistency in PCOS diagnosis and prevalence reporting.

**Abstract:**

Background: Newly named Polyendocrine Metabolic Ovarian Syndrome (PMOS), previously Polycystic Ovary Syndrome (PCOS), affects women worldwide. The prevalence of PCOS is thought to range from 5 to 15% depending on the diagnostic criteria used. The 2023 International Evidence-Based Guidelines for the Assessment and Management of PCOS recognize the 2003 Rotterdam criteria as the gold standard diagnostic tool. The objective was to examine the global prevalence of PCOS across diagnostic criteria, phenotypes, geographical locations, races, and ethnicities by reviewing current research. Methods: Researchers searched PubMed, PsycINFO, and Scopus. The study included 27 articles. Results: Prevalence varies globally, ranging from 1.6% to 28.5% in North America. In Brazil, the prevalence was 30.4%. Europe showed rates ranging from 5.9% to 19.9%. In Asia, the prevalence ranged from 4.21% in rural India to 35.3% in the Kashmir Valley. Oceania showed a prevalence of 6.8% among Samoan women and 12% in Australian women. The Middle East ranged from 1.6% in Dubai to 48.5% in Pakistan. In Africa, the prevalence was 32.5% in Sudan. Conclusions: The study’s findings highlight significant variations in the prevalence and phenotype distributions influenced by demographic factors and diagnostic criteria. Limitations include inconsistent diagnostic criteria, underrepresentation of geographical regions, and reliance on self-reported data, emphasizing the importance of using standardized diagnostic criteria and focused research.

## 1. Introduction

Polycystic ovary syndrome (PCOS), which was renamed in May 2026 to polyendocrine metabolic ovarian syndrome (PMOS), is not only a reproductive condition but also one of the most common endocrine disorders affecting females worldwide, with evidence suggesting that both external and internal factors, including environmental exposures, stress, genetics, lifestyle, and prenatal influences, play a role in its development [1]. This nomenclature change followed a rigorous, formally published international consensus process involving patients, multidisciplinary health professionals, and organizations across world regions [2]. The renaming reflects concerns that PCOS overemphasized ovarian cysts, which are not a defining feature of the condition, while obscuring its broader endocrine, metabolic, and reproductive impact. The consensus group recommended PMOS to more accurately represent the multisystem nature of the disorder while reducing stigma and improving communication, diagnosis, research, and clinical care. Although this terminology reflects an international consensus recommendation, full implementation is expected by the next international guideline update in 2028. Both PCOS and PMOS currently coexist in the scientific literature during this transition period. This manuscript retains “PCOS” throughout for consistency with existing research and terminology in use at the time of writing.

The pathophysiology and etiology of PCOS remain unclear, though several theories have been proposed regarding the underlying mechanism. However, the pathogenesis of polycystic ovary syndrome is thought to be multifactorial, with both hormonal and metabolic implications [3]. Androgens (male sex hormones), follicle-stimulating hormone, estrogen, luteinizing hormone, gonadotropin-releasing hormone, Anti-Müllerian hormone (AMH), and insulin are thought to be imbalanced in PCOS [4]. Characteristics of PCOS include hyperandrogenism, oligo or anovulation, and ovarian cysts. PCOS has been associated with many complications that can impact a person’s overall well-being and quality of life. PCOS has been associated with not only reproductive outcomes but also metabolic outcomes. PCOS has been associated with adverse health conditions like obesity, diabetes, dyslipidemia, cardiovascular disease, sleep apnea, nonalcoholic fatty liver disease, and depression [5]. Due to hyperandrogenism, women may experience conditions such as hirsutism, acne, acanthosis nigricans, infertility, endometrial cancer, and late menopause.

Clinical practice in assessing and managing PCOS tends to be inconsistent due to several different diagnostic criteria and a lack of a gold standard. The three diagnostic criteria used are [6]:

- The 1990 National Institute of Health (NIH) criteria;
- The 2003 Rotterdam criteria;
- The 2006 Androgen Excess and Polycystic Ovary Syndrome (AE-PCOS) Society criteria.

The three diagnostic criteria mentioned above differ slightly in biological, clinical, and image-based parameters for diagnosing (Table 1). The 2012 NIH Evidence-Based Methodology Workshop Panel, the 2018 Evidence-Based International Guidelines, and most recently the 2023 International Evidence-Based Guidelines have all contributed to the ongoing refinement of PCOS diagnostic criteria. This continuous evolution of diagnostic criteria illustrates how difficult it is to establish a stable prevalence estimate for PCOS. Nevertheless, the 2023 International Evidence-Based Guidelines for the Assessment and Management of PCOS recognize the Rotterdam criteria as the gold standard diagnostic tool for the diagnosis of PCOS, as it allows diagnosis across four distinct phenotypes [1]. Additionally, the 2023 International Evidence-Based Guidelines for the Assessment and Management of PCOS recognizes AMH as a biomarker for defining polycystic ovarian morphology in adults [1]. AMH is produced by granulosa cells of small antral and pre-antral follicles. It tends to be more elevated in women with PCOS due to having a greater number of small antral follicles present. So, having more of these follicles means that more total AMH is secreted into circulation [7]. However, there are pros and cons to using AMH as a biomarker to diagnosing polycystic ovarian morphology in women with PCOS. The pros of using AMH include that it is a non-invasive and objective measurement, that it correlates well with follicle count, that it is more consistent compared to other hormones since it fluctuates less across the menstrual cycle, and lastly, that it can be useful in women with obesity, where it might be harder to interpret ultrasound findings [7]. The cons of using AMH include that no standardized cut-off value exists, different immunoassay platforms produce different values, it naturally declines with age and varies by ethnicity and body weight, elevated AMH can occur in other conditions, and most data supporting the use of AMH comes from the Middle East and Asia, with notable gaps in research in European countries and the United States. These limitations underscore that AMH, much like diagnostic criteria for PCOS, is still evolving and inconsistently applied. This challenge may be reflected in the wide variability in PCOS prevalence worldwide.

The prevalence of PCOS is thought to range from 5 to 15% depending on the diagnostic criteria used, and about 75% of women remain undiagnosed due to the heterogeneity of patient presentation and lack of provider knowledge [8,9]. The different prevalence estimates are based on how restrictive each diagnostic tool is. The Rotterdam criteria require only 2 of 3 features and therefore allows for diagnosis without hyperandrogenism, capturing phenotype D in addition to the phenotypes recognized by the other criteria [8,9]. The AE-PCOS mandates hyperandrogenism as an obligatory feature, which excludes phenotype D and yields a narrower prevalence estimate than Rotterdam. The NIH criteria are the most restrictive and require both hyperandrogenism and oligo-/chronic anovulation to be present (Table 1). The NIH criteria would exclude phenotype C and D. The NIH criteria has the lowest reported prevalence among the three criteria sets, with pooled estimates of approximately 6% under NIH criteria compared to approximately 10% under Rotterdam and AE-PCOS criteria [10].

Currently, there are four phenotypic forms of PCOS: classic (A), incomplete classic (B), ovulatory (C), and non-androgenic (D) (Table 2). Phenotype A presents as oligo-anovulation, hyperandrogenism, and polycystic ovarian morphology. Phenotype B is when hyperandrogenism and oligo/anovulation are present. Phenotype C is defined as having hyperandrogenism and polycystic ovarian morphology, and lastly, phenotype D is oligo/anovulation and polycystic ovarian morphology [9]. The geographical location, region, and race play a role in PCOS phenotypic distribution and the different metabolic profiles of non-androgenic and androgenic phenotypes in various populations.

The research article “Geographical Prevalence of Polycystic Ovary Syndrome by Region, Race, and Ethnicity” examined the prevalence of PCOS using data from 2013 to 2015 [8]. Since then, very few studies have explored the prevalence of PCOS across different geographical regions, races, and ethnicities. This literature review aimed to assess the prevalence of PCOS across women in different geographical areas, races, and ethnicities by analyzing the most current and relevant research available.

## 2. Materials and Methods

### 2.1. Search Strategy and Selection Criteria

A protocol was developed before conducting the review, and an expert systematic review librarian was consulted to assist with the search strategy. The literature review used the PICO framework to examine the prevalence of PCOS among reproductive-aged females, with a particular emphasis on geographical location, race, and ethnicity, by reviewing relevant published studies. A comprehensive search was conducted to address this objective, focusing on studies published between 2018 and 2024. Searches were performed using electronic databases, PubMed, PsycINFO, and Scopus on 24 April 2024. The search strategy included using the terms ‘polycystic ovary syndrome’ and ‘prevalence’ combined using ‘AND.’ The search was limited to studies published between 1 January 2018 and 24 April 2024 (see Appendix A, Search Strategy in Table A1). Reference listing search was used as a supplemental search strategy. The Rayyan web application was used as a management tool to support the literature screening process.

The study’s inclusion criteria were publications translatable into English, studies published between 1 January 2018 and 24 April 2024, and research that addresses the prevalence of PCOS concerning geographical location and race/ethnicity in females aged 15–57 years old. This age range was chosen because it represents the reproductive years for most women, during which PCOS can have significant health implications. The exclusion criteria included studies published before 2018 and included participants less than 15 years old and/or greater than 57 years old. Gray literature searching was not conducted for this literature review.

### 2.2. Data Extraction and Analysis

Article duplicates were removed first before the titles and abstracts were screened for suitability. After the screening process, reviewers retrieved the screened articles meeting the inclusion criteria for a full-text review to validate the eligibility of studies to be included in the literature review. Discrepancies were settled through discussion.

The authors extracted relevant data from the studies that met the eligibility criteria. The key variables examined and extracted included authors, geographical location, study design, age range, prevalence estimate, and diagnostic criteria used. The authors developed a data extraction form to capture the variables and organize data. Due to the studies varying significantly and the use of different diagnostic criteria for PCOS, a statistical analysis was not conducted.

## 3. Results

### 3.1. Literature Search

**Figure A1:**
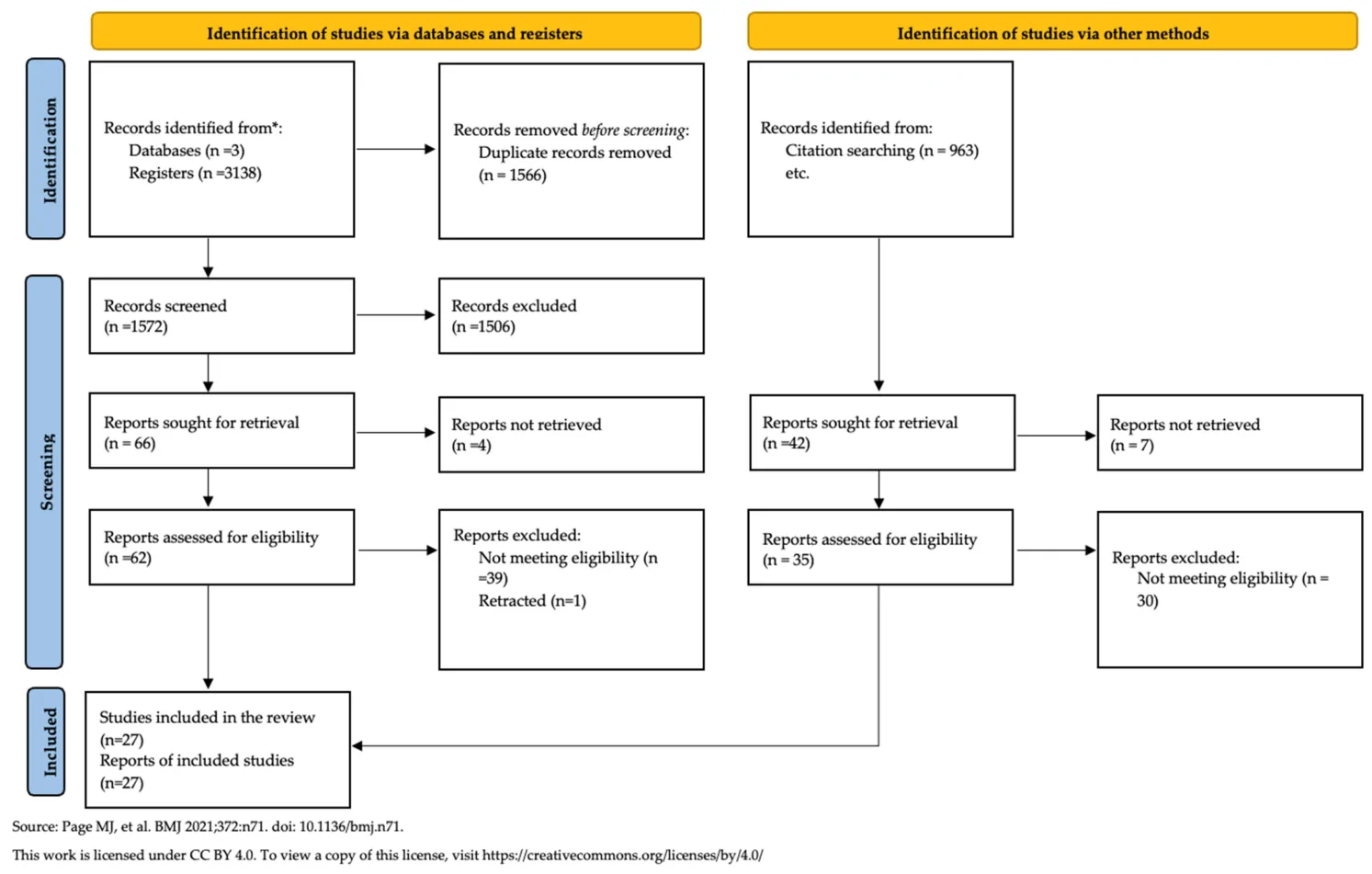
Prisma flow diagram [40].

A total of 3138 articles were identified from the three databases Scopus, PubMed, and PsycINFO. There were 2078 articles identified in Scopus, 874 identified from PubMed, and 186 identified in PsycINFO. There were 1566 articles that were duplicates and removed before the screening process, leaving 1572 articles for authors to screen based on the predefined inclusion criteria. After the screening process, 66 articles were retrieved; however, authors were unable to retrieve 4 articles in full-text format. Of the 62 articles assessed for eligibility, 23 met the inclusion criteria. An additional 5 articles identified through citation searching also met the inclusion criteria. One article was removed from the final analysis because it had been retracted. Therefore, a total of 27 articles were included (see Appendix A, PRISMA flow diagram in Figure A1).

### 3.2. Prevalence of PCOS by World Region

**Figure A2:**
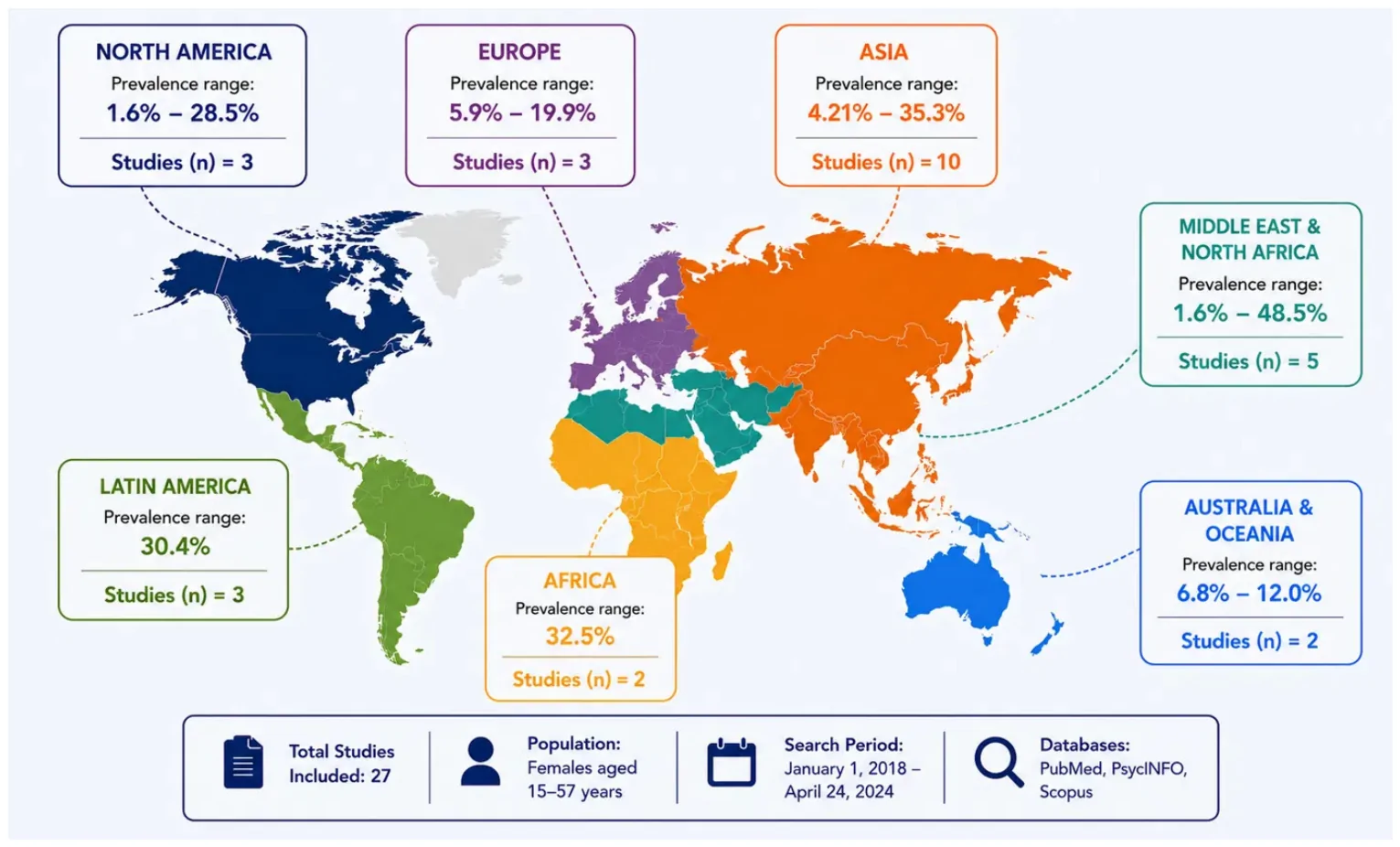
Geographical prevalence of PCOS by world region.

The prevalence of PCOS varied substantially across world regions (see Appendix A, Geographical prevalence of PCOS by world region in Figure A2).

#### 3.2.1. Prevalence of PCOS Across North America

Research examining the prevalence of PCOS across North America, including the United States (US) and Canada, remains limited (Table 3). The most current study looking at the prevalence in the US was a retrospective cohort study published in 2023 with 2491 participants. Results reported the prevalence of PCOS was 5.2% (2019) [11]. Investigators also concluded that the overall prevalence of PCOS was the highest among Hawaiian and Pacific Islanders, followed by Native Americans and then Hispanics [11]. This may suggest that Native Americans have a more severe phenotype when compared to other ethnic groups. Chiaffarino et al. conducted a meta-analysis and found that the prevalence of PCOS in the US was 6.2% using the NIH 1990, 20.7% using the Rotterdam criteria 2003, and 1.6% using ICD-9. Comparably, US females had a higher prevalence of phenotype A and lower phenotype C than European females. The study reported that the US and Europe had similar prevalence, which could be related to both being high-income areas; however, researchers concluded that more research is needed to determine the impact of economic influences [12]. Importantly noted is that different diagnostic criteria were used to determine the prevalence of PCOS in these studies examining the prevalence of PCOS in the US. Rao et al. sent out a questionnaire to women located in Dallas, Texas, where 28.5% of the respondents reported being formally diagnosed with PCOS [13]. The respondents of the survey were primarily Caucasian (68%), followed by Hispanics (15.2%), and then African Americans (10.5%).

#### 3.2.2. Prevalence of PCOS Across Latin America

Research has suggested that Hispanic women have a significantly higher prevalence of hirsutism, abnormal free androgen index, abnormal homeostasis model assessment, and hyperglycemia when compared to non-Hispanic women. A systematic review looking at the metabolic features of women with PCOS in Latin America found that in most studies, Latin women’s body mass index (BMI) fell in the overweight or obese category, and metabolic syndrome components like central obesity, low high-density protein (HDL), and hypertension were universal in women with PCOS from different Latin American countries [14]. There were a total of 6 studies looking at the prevalence of phenotypes. These studies were completed in Argentina, Brazil, and Chile. Phenotypes A and B were the most prevalent [14]. In a cross-sectional study by Tavares et al., researchers examined the prevalence of phenotypes in 111 women diagnosed with PCOS in Brazil [15]. Fifty-four percent of women met the criteria for phenotype A, 11.7% met the criteria for phenotype B, 14.4% met the criteria for phenotype C, and 19.8% for phenotype D [15]. Spritzer et al.’s meta-analysis looked at 12 articles that met eligibility criteria, which totaled 995 women with PCOS and 2275 control women, making the prevalence 30.4% [16]. A strength of this comparison was that all studies used the same diagnostic criteria—the Rotterdam criteria [16]. These women tended to have a higher BMI, waist circumference, blood pressure, glucose, Homeostatic Model Assessment of Insulin Resistance, and lipid profile than women without PCOS in Brazil [16]. Table 4 summarizes the design and findings of studies examining PCOS prevalence and phenotype distribution across Latin America.

#### 3.2.3. Prevalence of PCOS Across Europe

There was limited research studies examining PCOS across Europe (Table 5). In Chiaffarino et al.’s research article, they reported the prevalence of PCOS in northern Europe was 6.6% using the NIH criteria, 18.3% using the Rotterdam criteria, and 14.9% using the 2006 AE-PCOS criteria [12]. The prevalence of PCOS in southern Europe was 5.9% using NIH criteria, 15.3% using the 2006 AE-PCOS criteria, and 19.9% using the Rotterdam criteria [12]. As already mentioned, high-income European countries and the United States have a similar prevalence of PCOS. Europe and the United States had a higher prevalence of phenotype A in all considered areas [12]. Still, the prevalence of phenotype D was higher in northern/central Europe than in other regions [12]. Tararchuk et al. conducted a cross-sectional study that examined records of obstetrician-gynecologists who were a part of the Ukrainian Society of Gynecological Endocrinology patients’ medical records diagnosed with PCOS by the Rotterdam criteria [17]. Phenotype A was the most prevalent (47.7%), whereas phenotypes B (17.6%), C (17.4%), and D (17.3%) were equally distributed. The study found that women with phenotype A had the highest BMI, followed by phenotype B and D [17]. Phenotype C had the lowest BMI. Hirsutism was present in 57.6% [17]. In a retrospective study by Carmina et al. in the Mediterranean, researchers examined the prevalence of PCOS phenotypes in a Sicilian population of women diagnosed with PCOS using the Rotterdam criteria. Out of the 1215 women, 57.7% had phenotype A, 9.2% had phenotype B, 30% had phenotype C, and 3.1% had phenotype D [18].

**Table 3:** Summary of studies on the prevalence of PCOS in North America.

Study Author	Study Design	Location	Age Range	Prevalence	Limitations
Yu et al. [11]	Retrospective cohort	United States	16–40 years old	5.2% was the prevalence based on 3036 of 58,214 identified cases of female patients in 2019	Used ICD-9 or ICD-10 codes to identify PCOS Only examined insured patients in the United States 73% of enrollees were non-Hispanic white
Chiaffarino et al. [12]	Meta-analysis	United States	18–49 years old	6.2% was the prevalence of PCOS based on NIH 1990 criteria 20.7% was the prevalence of PCOS based on the Rotterdam criteria 1.6% was the prevalence of PCOS based on ICD-9 codes The United States had a higher prevalence of phenotype A and a lower prevalence of phenotype C compared to Europe	Researchers examined only 8 studies from the USA Only one study examined the prevalence of PCOS in the United States using the Rotterdam criteria
Rao et al. [13]	Cross-sectional study	United States	Mean age 32.9 ± 11.4 years	28.5% of female respondents indicated a formal diagnosis of PCOS 40.5% of female respondents thought to have PCOS without a formal diagnosis, but had 2 or more symptoms aligning with PCOS	There were variations in diagnostic criteria Potential measurement bias due to researchers relying on self-reported data

**Table 4:** Summary of studies on the prevalence of PCOS in Latin America.

Study Author	Study Design	Location	Age Range	Prevalence	Limitations
Marchesan et al. [14]	Systematic Review	Brazil	21–30 years old	Phenotypes A and B were the most prevalent Phenotype A ranged from 33.3 to 44% Phenotype B ranged from 15 to 58% Phenotype C ranged from 11.9 to 36% Phenotype D ranged from 14.2 to 66%	Relatively few studies have found that despite the vast size of the region There could be possible heterogeneity due to the small sample sizes and lack of studies, small sample, and lack of studies in some regions of the world
Tavares et al. [15]	Cross-sectional study	Brazil	18–39 years old	54% meet the criteria for Phenotype A 11.7% meet the criteria for Phenotype B 14.4% meet criteria for Phenotype C 19.8% meet the criteria for Phenotype D	Only evaluated hyperandrogenism by evaluating total blood levels of testosterone; however, the measurement of free testosterone or free testosterone index is the most sensitive measure to assess hyperandrogenism Researchers only considered an ovarian volume > 10 cm when evaluating ultrasound scans and were not able to obtain follicle counting information, which is recommended by the Rotterdam criteria
Spritzer et al. [16]	Meta-analysis	Brazil	18–39 years old	30.4% was the prevalence according to the Rotterdam criteria, with 995 women having PCOS and 2275 women acting as the control group	Included a small number of studies, small sample sizes, and heterogeneity across the studies

**Table 5:** Summary of studies on the prevalence of PCOS in Europe.

Study Author	Study Design	Location	Age Range	Prevalence	Limitations
Chiaffarino et al. [12]	Meta-analysis	Northern Europe Southern Europe	18–49 years old	6.6% was the prevalence using the NIH criteria, 18.3% was the prevalence using the Rotterdam criteria, 14.9% was the prevalence using the 2006 AE-PCOS criteria in northern Europe 5.9% was the prevalence using the NIH criteria, 19.9% was the prevalence using the Rotterdam criteria, 15.3% was the prevalence using the 2006 AE-PCOS criteria in southern Europe Higher prevalence of phenotype A in all considered areas There was a higher prevalence of phenotype D in northern and central Europe compared to other areas in Europe	The use of different diagnostic criteria Studies may have varying clinical cut-offs for diagnosing PCOS Lifestyle factors, body composition, and hormonal therapy were not controlled for and could lead to inaccuracies in determining the prevalence
Tarachuck et al. [17]	Cross-sectional study	Ukraine	20–45 years old	47.7% were diagnosed with phenotype A 17.6% were diagnosed with phenotype B 17.4% were diagnosed with phenotype C 17.3% were diagnosed with phenotype D	Focused on a selected population (referral population) and may not represent the true prevalence of PCOS phenotypes in the general Ukrainian population Mild biochemical hyperandrogenism may have been missed due to the study focusing on mandatory biochemical assessment for hyperandrogenism Potential misdiagnosis of PCOS phenotypes in underweight individuals, causing some cases to be incorrectly categorized The study’s cross-sectional design limits the ability to conclude the natural progression of PCOS phenotypes with age
Carmina et al. [18]	Retrospective study	Sicily	18–40 years old	57.5% of women had phenotype A 9.2% of women had phenotype B, and concluded this may present the worst metabolic profile 30% of women had phenotype C 3.1% of women had phenotype D	The retrospective nature No detailed data on lifestyle and diet

#### 3.2.4. Prevalence of PCOS Across Asia

The highest number of studies focusing on the prevalence of PCOS was in Asia (Table 6). Yang et al. completed a comprehensive survey of 12,815 women aged 20–49 years old. Eight hundred and twenty-six of those women were diagnosed with PCOS according to the Rotterdam criteria. The weighted prevalence rate was 7.8% [19]. Researchers using age-specific prevalence rates estimated that there are 24 million women with PCOS in China. They also saw an increase in prevalence from 5.6% in 2010 to 8.6% in 2022 and an increase in overweight women and having polycystic ovaries when compared to 2010 [19]. In a cross-sectional study, Bigambo et al. examined menstruation and gynecological disease prevalence among Chinese women. They reported the prevalence of PCOS to be 19.04% in their sample of 977 women aged 18–55 years old [20].

In a group of 544,619 women aged 15–49 years old in Korea, the prevalence of PCOS was 4.3% from 2010 to 2019 using the Korean Informative Classification of Disease, version 10 ICD-codes E.28-E28.9 in the population-based National Health Information Databases, which includes all insurance claims for approximately 97% of the population in Korea [21]. In another cross-sectional study, researchers examined the prevalence of PCOS in women aged 18–29 years old at the University in Korea. The prevalence of PCOS was 5.2% based on the NIH criteria [22].

Another study looked at the prevalence of PCOS in the Klang Valley. Most respondents were Chinese (68.5%), followed by Malays (16.1%), Indians (12.4%), and lastly, 2.9% were other ethnicities. According to the survey, 10.49% were medically diagnosed with PCOS, 2.9% were diagnosed with PCOS based on signs and symptoms, and 32.92% were suspected of having PCOS [23]. A cross-sectional study conducted by Dashit et al. looked at the prevalence of PCOS in females working at the University Putra Malaysia. The prevalence was reported as 12.6% using the Rotterdam criteria [24]. Interestingly, all participants had elevated levels of total and free testosterone and a positive ultrasound finding of polycystic ovaries. Only 1.2% of participants had anovulation [24]. Cao et al. conducted a cross-sectional study looking at the prevalence of phenotypes. Phenotype D was the most prevalent (67.6%) followed by phenotype A (16.7%) then phenotype C (12.7%) and B (3.1%) [25].

The Kashmir Valley is a region in northern India whose population reflects a mix of Indigenous, Central Asian, and Mughal ancestry. A cross-sectional study examined the prevalence of PCOS among Kashmiri women and found the prevalence to be 28.9% with the NIH criteria, 35.3% according to the Rotterdam criteria, and 34.3% according to the 2006 AE-PCOS criteria [26]. The highest prevalence of PCOS was among women 20–29 years old. Fifty-two percent had oligomenorrhea, hyperandrogenism, and polycystic ovary morphology, 23.7% had hyperandrogenism with polycystic ovary morphology, 22.1% had oligomenorrhea with hyperandrogenism, and 3.0% had oligomenorrhea with polycystic ovary morphology [26]. In another cross-sectional study conducted by Deswal et al., researchers observed the prevalence of PCOS in Haryana, India, with both rural and urban populations [27]. This study concluded the prevalence of PCOS was 4.21% using the Rotterdam criteria [27]. Most women diagnosed with PCOS were from urban areas (71%) compared to rural areas (29%). Twenty-four out of 94 of the participants (26%) had menstrual irregularities, hyperandrogenism, and polycystic ovaries [27]. Gupta et al. completed a cross-sectional study in females aged 15–24 years. The prevalence of PCOS was 22.85% by the Rotterdam criteria and 10.7% according to the 2006 AE-PCOS criteria [28].

**Table 6:** Summary of studies on the prevalence of PCOS in Asia.

Study Author	Study Design	Location	Age Range	Prevalence	Limitations
Yang et al. [19]	Cross-sectional study	China	20–49 years old	In total, 7.8% was the prevalence of PCOS according to the Rotterdam criteria Prevalence of PCOS decreased with age, ranging from 14.7% in women aged 20–24 years to 1.2% in women aged 45–49 years old Prevalence of PCOS increased from 5.6% in 2010 to 8.6% in 2020	Sample bias due to only 8 out of 15 provinces covered in the 2010 survey were included Challenges in diagnosing PCOS in older women due to changes from menopause There was a lack of metabolic data from the 2020 survey Limited generalizability due to the sample consisting of the Han Chinese ethnicity
Bigambo et al. [20]	Cross-sectional study	China	18–55 years old	In total, 19.04% was the prevalence of PCOS according to a questionnaire asking about being diagnosed with gynecological diseases in its third section	The self-reported data can lead to recall bias regarding menstrual and disease history Findings may not be relevant to all Chinese women since the research was conducted at a single hospital in Nanjing, China
Kim et al. [21]	Cohort study	Korea	15–49 years old	In total, 4.3% was the prevalence of PCOS in Korean women aged 15–49 years old Women with PCOS had a higher BMI, fasting blood glucose, and lower HDL	Does not mention what diagnostic criteria were used to diagnose women with PCOS because Researchers looked at ICD-codes
Park et al. [22]	Cross-sectional descriptive correlational study	Korea	18–29 years old	In total, 5.2% was the prevalence of PCOS based on the NIH criteria Women with PCOS had significantly higher fat-free mass, muscle mass, and body fluid Had lower sex hormone binding globulin and higher free androgen index	Did not account for cofounding factors like Cushing Syndrome or congenital adrenal hyperplasia that can lead to hyperandrogenism Participants self-reported on irregular menstruation, which could lead to bias due to personal perception Volunteered for the study, which could result in menstrual issues being more motivated to participate in the study, resulting in a higher prevalence of menstrual issues
Goh et al. [23]	Descriptive cross-sectional study	Malaysia	18–45 years old	In total, 10.49% were medically diagnosed with PCOS based on the survey In total, 2.9% were diagnosed with PCOS based on signs and symptoms In total, 32.92% were suspected of having PCOS The study suggested Malaysians had a lower prevalence of PCOS	Underrepresentation of underserved populations due to being conducted through social media platforms Self-reported data, which could lead to bias Selection bias due to convenience sampling
Dashti et al. [24]	Cross-sectional study	Malaysia	18–49 years old	In total, 12.6% was the prevalence of PCOS among Malaysian Female University Staff using the Rotterdam criteria All participants had elevated levels of total and free testosterone and positive ultrasound findings of polycystic ovaries In total, 98% had hyperandrogenism and polycystic ovaries (phenotype C) In total, 1.2% had hyperandrogenism, polycystic ovaries, and anovulation (phenotype A)	May not fully represent the broader population of women with PCOS Not all participants underwent a blood test due to financial constraints
Cao et al. [25]	Cross-sectional study	Vietnam	18–45 years old	In total, 16.5% had phenotype A In total, 3.1% had phenotype B In total, 12.7% had phenotype C In total, 67.6% had phenotype D	Not generalizable to Southeast Asian populations Due to the nature of the cross-sectional study, researchers are only able to evaluate metabolic risk factors at a single point in time
Ganie et al. [26]	Cross-sectional study	India	15–40 years old	In total, 28.9% was the prevalence using the NIH criteria In total, 35.3% was the prevalence using the Rotterdam criteria In total, 34.3% was the prevalence of PCOS among women 20–29 years old In total, 52.6% had oligomenorrhea, hyperandrogenism, and polycystic ovary morphology In total, 23.7% had hyperandrogenism and polycystic ovary morphology In total, 22.1% had oligomenorrhea and hyperandrogenism In total, 3.0% had oligomenorrhea with polycystic ovary morphology	May not represent the general population since it was conducted in educational institutes Participants volunteered, which could result in over-representation of symptomatic women with PCOS and lead to a higher prevalence
Deswal et al. [27]	Random large-scale observational cross-sectional study	India	16–45 years old	In total, 4.21% had the prevalence of PCOS according to the Rotterdam criteria In total, 26% had menstrual irregularities, hyperandrogenism, and polycystic ovaries Women living in urban areas had a higher prevalence of PCOS	The study is only generalizable to individuals living in Haryana
Gupta et al. [28]	Cross-sectional study	India	15–24 years old	In total, 22.85% was the prevalence of PCOS diagnosed with the Rotterdam criteria 10.7% was the prevalence of PCOS diagnosed with the 2006 AE-PCOS criteria	Reports on the prevalence of PCOS using the Rotterdam and 2006 AE-PCOS criteria Low participation and sampling bias Potential misclassification due to inconsistent free testosterone testing

#### 3.2.5. Prevalence of PCOS Across Australia and Oceania

In a sub-study by Varanasi et al., researchers examined the prevalence of PCOS individuals 16–29 years old living in Victoria, Australia [29]. Researchers reported the prevalence of PCOS in their cohort was 12% according to the NIH criteria [29]. Maredia et al. performed a cross-sectional study examining the relationship of PCOS among Samoan women. They found the prevalence to be 6.8% based on the NIH criteria [30]. Women with PCOS had a higher BMI, percent body fat, abdominal circumference, triglycerides, fast insulin, prevalence of insulin resistance, total testosterone, free androgen index, and anti-Mullerian hormone [30]. Table 7 summarizes the design and findings of studies examining PCOS prevalence and phenotype distribution across Australia and Oceania.

#### 3.2.6. Prevalence of PCOS Across the Middle East

A few studies examined the prevalence of PCOS across the Middle East (Table 8). Mousa et al. conducted a meta-analysis examining the prevalence of PCOS across the Middle East [31]. Twenty-six studies were included in the analysis using the NIH criteria, 15 studies using the Rotterdam criteria, and three studies using both the NIH and Rotterdam criteria. The studies concluded the population-based pooled prevalence of PCOS in the Middle East was 8.9% according to the NIH criteria and 11.9% according to the Rotterdam criteria [31].

Mirza et al. conducted a cross-sectional study that reviewed medical records of women 15 to 45 years old from 2017 to 2022 at Latifa Hospital, Dubai [32]. The prevalence of PCOS was 1.6% among the 64,722 females whose medical charts were reviewed between 2017 and 2022 [32]. The study found the prevalence of PCOS rose from 1.19% in 2020 to 2.72% in 2022 [32]. Memon et al. conducted a cross-sectional study examining the Nazeer Hussain Medical Complex with 200 participants using the Rotterdam criteria in women aged 16 to 40. The prevalence of PCOS was 48.5% in this population [33]. A cross-sectional study conducted in Saudi Arabia reported the prevalence of PCOS as 32.5% [34].

A retrospective cross-sectional study conducted in Lebanon reviewed the prevalence of phenotypes in women aged 15–44 years old diagnosed with PCOS using the 2003 Rotterdam criteria [35]. The sample included 322 Lebanese women. Phenotype A, classified as oligo-or anovulation with hyperandrogenism and polycystic ovaries, was the most prevalent and present in 67% of the sample. Phenotype D was the second most common phenotype in 15% of the women. In total, 12% had phenotype C, and 6% had phenotype B [35].

#### 3.2.7. Prevalence of PCOS Across Africa

Africa is the second-largest and second-most-populous continent in the world, with many countries within the region continuing to face significant economic and healthcare challenges. However, research examining the prevalence of PCOS in this area is limited (Table 9). Alfanob et al. performed a descriptive cross-sectional community-based study looking at the prevalence of PCOS in Khartoum State, Sudan, among reproductive-aged women [36]. They concluded the prevalence of PCOS in this sample was 32.5% [36]. Elasam et al. looked at the phenotype expression in a cross-sectional study, a descriptive study in infertile women with PCOS based on the Rotterdam criteria in Khartoum, Sudan [37]. Researchers found that phenotype D was the most prevalent (51.6%), followed by phenotype B (22.6%), phenotype C (18.2%), and phenotype A (7.6%) [37].

**Table 7:** Summary of studies on the prevalence of PCOS in Australia and Oceania.

Study Author	Study Design	Location	Age Range	Prevalence	Limitations
Varanasi et al. [29]	Cross-sectional study	Australia	16–29 years old	In total, 12% was the prevalence of PCOS according to the NIH criteria	The use of the NIH criteria may be less comprehensive in certain cases compared to other diagnosis criteria Self-reported PCOS was not confirmed with medical records and could lead to recall bias Had a small sample size Serum androgen levels were not available for all individuals, which limits the ability to accurately apply NIH diagnostic criteria
CMaredia et al. [30]	Cross-sectional study	Samoa	25–39 years old	In total, 6.8% was the prevalence of PCOS based on the NIH criteria Polycystic ovary + Hyperandrogenism was the most common (phenotype c) Oligomenorrhea/anovulation + hyperandrogenism and Oligomenorrhea/anovulation + Hyperandrogenism + Polycystic ovary had the greatest degree of metabolic abnormality	Used self-reported data that can lead to recall bias, errors in translation, and variations in survey administration Lacked clinical examination of hyperandrogenism Lacked true clinical under-or overestimates

**Table 8:** Summary of studies on the prevalence of PCOS in the Middle East.

Study Author	Study Design	Location	Age Range	Prevalence	Limitations
Mousa et al. [31]	Meta-analysis	Middle East	Age mean 15.2–56.5 years old	In total, 8.9% was the pooled prevalence of PCOS according to the NIH criteria In total, 11.9% was the pooled prevalence of PCOS according to the Rotterdam criteria	Diagnostic criteria were not standardized Possible selection bias due to many studies relying on retrospective screening or convenience sampling Many studies included low methodological quality, which undermines
Mirza et al. [32]	Cross-sectional study	United Arab Emirates	15–45 years old	In total, 1.6% was the annual prevalence of PCOS from 2017 to 2022 using ICD-10 codes E28.2 and Z87.2 Prevalence of PCOS rose 1.19% in 2020 to 2.72% in 2022	Potential misclassification and inaccuracies due to the use of electronic medical and ICD-10 codes Lack of confirmation with the clinical test COVID-19 could have changed the prevalence Limited generalizability
Memon et al. [33]	Cross-sectional study	Pakistan	16–40 years old	In total, 48.5% was the prevalence of PCOS according to the Rotterdam criteria	Potential measurement bias due to relying on self-reported data for certain aspects of the study
Alraddadi et al. [34]	Cross-sectional study	Saudi Arabia	18–45 years old	In total, 32.5% was the prevalence of PCOS	Lack of objective diagnosis of PCOS Reliance on self-reported data
Naous et al. [35]	Cross-sectional study	Lebanon	15–44 years old	In total, 67% of women had phenotype A In total, 15% of women had phenotype D In total, 12% of women had phenotype C In total, 6% had phenotype B	The collected data was based on a self-reported questionnaire Participants were recruited from outpatient clinics and may not apply to those with milder phenotypes

**Table 9:** Summary of studies on the prevalence of PCOS in Africa.

Study Author	Study Design	Location	Age Range	Prevalence	Limitations
Alfanob et al. [36]	Cross-sectional study	Sudan	16–50 years old	In total, 32.5% of participants taking the questionnaire reported having PCOS in Khartoum	Due to the nature of the cross-sectional study, researchers were unable to establish the temporal relationship with risk factors Should not be considered representative of Sudan since participant recruitment only took place in the Khartoum state
Elasam et al. [37]	Cross-sectional Study	Sudan	18–45 years old	In total, 51.6% of the participants had phenotype D using the Rotterdam criteria In total, 22.6% of the participants had phenotype B using the Rotterdam criteria In total, 18.2% had phenotype C using the Rotterdam criteria In total, 7.6% had phenotype A using the Rotterdam criteria Phenotype D was the most prevalent	Conducted in infertile women only and may not represent the broader PCOS population in Sudan

## 4. Discussion

The review focused on investigating the prevalence of polycystic ovary syndrome (PCOS) across various geographical regions and among different racial and ethnic groups. It aimed to present regional differences in the prevalence of PCOS based on different populations worldwide. These regions included North America, Latin America, Europe, Asia, the Middle East, and Africa. Additionally, ethnic, metabolic, and lifestyle factors were also shown to influence the prevalence of PCOS in studies. Findings from the study showed regional variations influenced by the varying diagnostic criteria, even with the evidence-based international guidelines strongly recommending the use of the Rotterdam criteria. Salari et al. recently published a systematic review and meta-analysis of PCOS’s global prevalence. Researchers reported the global prevalence of PCOS as 9.2% [38]. The publication dates of the studies included in the systematic and meta-analysis ranged from 1999 to 2020. Salari et al. included 31 studies conducted in Asia, five in Europe, two in Africa, six in America, and four in Australia. They concluded that the prevalence of PCOS was 9.8% in Asia, 14.2% in Europe, 3.3% in America, 16.4% in Africa, and 12.9% in Australia [38]. Yasmin et al. published a review looking at the prevalence of PCOS in 2022; however, it reported on very similar findings to those in Wolf et al.’s literature review [8,9].

This review examined articles published from 2018 to 2024 to gather the most recent data. Unlike other studies, researchers chose to investigate studies conducted in the Middle East separately from those in other Asian countries and to explore the prevalence of PCOS in Latin America apart from North America. In addition to evaluating the overall prevalence of PCOS, the review included articles discussing the prevalence of various phenotypes across different regions worldwide. A total of 27 articles were included in the study: three focused on North America, three on Latin America, three on Europe, ten on Asia, two on Australia and Oceania, five on the Middle East and Northern Africa, and two on Africa. Interestingly, there were no articles examining the prevalence of PCOS in Canada. The overall prevalence of PCOS in North America ranged from 1.6% to 28.5% [12,13]. Notably, only one study in this literature review examined the overall prevalence of PCOS in Brazil, reporting a rate of 30.4% [16]. Two studies focused on the prevalence of PCOS phenotypes, with findings indicating that phenotype A was the most prevalent [14,15]. The prevalence of PCOS in Europe varies across different regions. In northern Europe, studies report rates ranging from 6.6 to 18.3% [12]. In southern Europe, the rates ranged from 5.9 to 19.9% [12]. Phenotype A is the most prevalent form in all three studies. Additionally, research conducted in Ukraine and Sicily revealed similar trends, with 47.7% of women in Ukraine and 57.7% in Sicily exhibiting phenotype A [17,18]. The prevalence of PCOS varies across different countries and populations throughout Asia, with rates ranging as low as 4.21% in rural India to as high as 35.3% in the Kashmir Valley [26,27]. The diagnostic criteria and the demographics being studied play a role in the prevalence of PCOS. In China, the weighted prevalence was 7.8%, with estimates indicating that 24 million women were affected [19]. There was a noticeable increase in prevalence from 5.6% in 2010 to 8.6% in 2022 [19]. In Korea, the prevalence ranged from 4.3% based on national health data using the NIH criteria to 5.2% in a university study [21,22]. Malaysia reports a prevalence of 10.49–12.6% [23,24]. In India, urban areas showed a higher prevalence, with some regions like the Kashmir Valley experiencing exceptionally high rates with a prevalence of 35.3% [26]. The prevalence of PCOS phenotypes also varied across different populations. For instance, in a Vietnamese study, phenotype D (oligomenorrhea and hyperandrogenism without polycystic ovaries) was the most common, affecting 67.6% of women, while phenotype A (all three criteria) was less common [25]. In the Kashmir Valley, phenotype A was the most prevalent, with 52.6% of women exhibiting all three major features of PCOS—oligomenorrhea, hyperandrogenism, and polycystic ovaries [26]. Other regions, such as Haryana, and among younger women in India, also show a higher prevalence of phenotype A [27]. In Australia and Oceania, the prevalence of PCOS ranged from 6.8% among Samoan women to 12% among Australian women aged 16-29 [29,30]. PCOS prevalence in the Middle East ranged from 1.6% in Dubai to 48.5% in Pakistan [32,33]. In Lebanon, phenotype A (oligo-anovulation, hyperandrogenism, and polycystic ovaries) was the most common, affecting 67% of women with PCOS [35]. The prevalence of PCOS in Sudan was 32.5%, with a study concluding that phenotype D was the most common [36,37].

Regarding the variation distribution of phenotypes across different world regions, the studies from the United States, Europe, Latin America, and the Middle East reported phenotype A to be the most common. The predominance of phenotype A may be due to it being the most symptomatic phenotype. It includes all three key features of PCOS, and women with this presentation may have more noticeable symptoms compared to women with the other phenotypes. Additionally, phenotype A qualifies for diagnosis under every diagnostic tool created, whereas other phenotypes like C and D can slip through the cracks depending on which diagnostic tool is being used in the study. However, some populations may have a higher prevalence of phenotype A because of androgen expression, insulin resistance, and overall body composition. One study in Africa (Sudan) and one in Asia (Vietnam) showed that women with PCOS predominantly had phenotype D, which is the non-androgenic phenotype. Phenotype C was the most predominant phenotype in another study in Asia, more specifically Malaysia. These regional variations are clinically significant because the phenotypes differ meaningfully in their associated health risks. The androgenic phenotypes are generally associated with worse metabolic disturbances, including insulin resistance and cardiometabolic risks, compared to non-androgenic phenotypes [39]. It is important for researchers, policymakers, and healthcare providers to know the most common phenotype in a region, because this can affect what care patients need. It is also important to understand that places where non-androgenic phenotypes are more common may have higher rates of underdiagnosis because the more visible androgen-related symptoms are absent. Relatively few studies reported phenotypic distribution, so these regional patterns should be interpreted with caution. Going forward, larger and more consistent reporting of phenotype data across regions is needed to confirm these trends.

A strength of the study is its analysis of the prevalence of PCOS across diverse global regions and ethnic groups, including studies from North America, Latin America, Europe, Asia, Oceania, the Middle East, and Africa. The review incorporates a broad selection of studies published between 2018 and 2024, ensuring the findings are current and relevant. The study also examines the prevalence and distribution of PCOS phenotypes, offering an understanding of regional variations. This illustrates how different factors such as diagnostic criteria and demographic characteristics shape PCOS rates.

Differences in diagnostic criteria, ethnicity, healthcare access, and population demographics likely contribute to the variation in reported PCOS prevalence across regions. The 1990 NIH criteria and 2006 AE-PCOS criteria tend to be stricter than the 2003 Rotterdam criteria as they do not capture all the phenotypes. This highlights the need for more standardized research studies using the Rotterdam criteria to clarify these differences and provide more accurate estimates.

Although this review aimed to include global representation of all studies meeting the inclusion criteria, the geographical distribution of included studies was markedly uneven. As expected, there was limited representation of certain global regions, especially Latin America, Oceania, and Africa. It is important to note that the imbalance limits the extent to which the findings can be generalized as a global picture of representation of PCOS prevalence. Most importantly, regions with fewer studies and underrepresentation can be a result of gaps in research infrastructure, funding, publication access, healthcare access, and awareness of PCOS rather than clear differences in disease prevalence. This does not necessarily mean that PCOS is less prevalent in these areas, and the reported prevalence from this review should be interpreted as reflective of the available peer-reviewed literature rather than precise global estimates. This limitation further highlights future needs and opportunities to focus on underrepresented and understudied geographical locations and regions around the world. There were relatively few studies that included phenotypic distribution and regional patterns should be interpreted with caution. Larger and more consistent reporting of phenotype data across regions is needed to confirm these trends.

Another limitation of this review is that a formal quality appraisal tool, such as the Newcastle-Ottawa Scale (NOS), was not conducted. The scope of this review was intentionally broad to provide a comprehensive overview of the available literature on PCOS prevalence across geographical locations and regions. The objective was to describe the current global body of evidence rather than provide quantitative prevalence estimates. Additionally, excluding studies based on a formal quality appraisal may have further reduced the representation of regions where prevalence data are limited, such as Africa and Oceania. This would have further limited the review’s ability to characterize the current global evidence and identify important gaps in the literature, including the underrepresentation of certain areas, inconsistent use of diagnostic tools, and limited reporting on phenotype distribution.

Some studies included in the review relied on indirect measures, such as self-reported data and ICD-9 codes, rather than direct clinical assessments. These approaches can underestimate the prevalence of PCOS and introduce potential biases, including symptom misclassification and inaccurate diagnosis reporting. Self-reported data may underestimate the prevalence of PCOS because women would have had to have been diagnosed with PCOS, which would miss cases where women may not have been diagnosed yet. ICD codes only count women who have been diagnosed and recorded in the healthcare system. This would leave out women who are undiagnosed or do not have easy access to healthcare. As a result, many underserved regions may have a lower prevalence of PCOS because fewer cases are captured. Lastly, healthcare providers may have different approaches to how they code PCOS and may record only presenting symptoms rather than the diagnosis itself. Thus, prevalence estimates based on the indirect measures mentioned above may underestimate the true prevalence of PCOS, and regional differences may be reflective of the measurement method used rather than actual disease burden.

Additionally, it is important to recognize there may be underdiagnosis of PCOS in underserved areas with limited access to healthcare. As mentioned, this disparity may impact who is captured in prevalence data. A reason for this can be limited access to diagnostic tools, such as biochemical tests and ultrasound, along with a lack of trained healthcare personnel and lower health literacy. These factors, along with differences across ethnic groups in body composition, insulin resistance, and androgen expression, drive regional variation in how PCOS presents and is diagnosed. Unfortunately, underestimation of its prevalence may cause PCOS-related health needs to be overlooked and underfunded in underserved regions that have the greatest need.

Recommendations for future research include conducting population-based prevalence studies in underrepresented regions, especially Africa, Oceania, and other areas with limited available data, such as Canada. Additionally, both future studies and clinical practice should adopt standardized diagnostic criteria, such as the Rotterdam criteria and current international evidence-based guidelines, to improve diagnostic consistency and comparability across studies and regions. Future research should also consistently report phenotype distributions and include diverse populations to improve understanding of global variation in the prevalence and phenotype distribution of PCOS. A recommendation for policymakers would be to improve healthcare access for underserved populations, along with clinical training and consistent diagnosis. This would not only improve the quality of life for women with PCOS but also improve both diagnostic accuracy and the reliability of data used to estimate the prevalence of PCOS. Another recommendation for policymakers would be to include PCOS in chronic disease policies in order to support more equitable care and early detection. Lastly, future systematic reviews and meta-analyses should incorporate standardized quality appraisal tools to strengthen methodological rigor and confidence in the findings.

## 5. Conclusions

The review provides a comprehensive analysis of PCOS by exploring its prevalence and phenotype distribution across different regions and ethnic groups. The findings highlight significant variations in PCOS prevalence and phenotype distribution influenced by diagnostic criteria and demographic factors. Across the 27 studies included, the prevalence of PCOS varied widely from 1.6 to 48.5% depending on the region and diagnostic criteria. Phenotype distribution also varied by region with phenotype A more predominant in some studies from the United States, Europe, Latin America, and the Middle East, while phenotype D was found to be more common in Vietnam and Sudan. Despite the 2003 Rotterdam criteria being the gold standard, most studies continue to rely on the 1990 National Institutes of Health criteria and the 2006 Androgen Excess and PCOS Society criteria when looking at the prevalence of PCOS. This may reflect limited adoption of current evidence-based guidelines and contribute to inconsistent estimates. It is essential to understand these regional variations because differences in prevalence and phenotype distribution can guide screening efforts and reduce underdiagnosis in underserved areas. While the study offers valuable insights into the global landscape of PCOS by region, it also has limitations. Key limitations included inconsistent diagnostic practices, underrepresentation of certain regions, and reliance on self-reported data, emphasizing the need for more standardized and rigorous research. Future studies should address these limitations using consistent diagnostic criteria and expanding data collection methods to achieve a more accurate and representative understanding of PCOS worldwide.

## Figures and Tables

**Table 1:** The three different diagnosis criteria for PCOS [8,9].

National Institutes of Health (NIH 1990)	Rotterdam 2003	2006 AE-PCOS Society Criteria
Need both criteria for diagnosis: Hyperandrogenism Oligo-or chronic anovulation	Need 2 of 3 criteria for diagnosis: Hyperandrogenism Oligo-and/or anovulation Polycystic-appearing ovarian morphology	Required for diagnosis: Hyperandrogenism Plus, either one of the following: Oligo-and/or anovulation Polycystic-appearing ovarian morphology
Attempted to produce a clinical definition of PCOS Excludes Phenotype C of PCOS	Broadened the phenotypic expression of PCOS by allowing the diagnosis of PCOS without hyperandrogenism	Wanted to provide an evidence-based definition Excludes phenotype D of PCOS

**Table 2:** The classification of PCOS phenotypes. Adapted from Yasmin et al. [9].

Phenotype A	Phenotype B	Phenotype C	Phenotype D
Hyperandrogenism Oligo/anovulation Polycystic ovarian morphology	Hyperandrogenism Oligo/anovulation	Hyperandrogenism Polycystic ovarian morphology	Oligo/anovulation Polycystic ovarian morphology

## Data Availability

The original contributions presented in this study are included in the article. Further inquiries can be directed to the corresponding author.

## Abbreviations

The following abbreviations are used in this manuscript:

PMOS	Polyendocrine Metabolic Ovarian Syndrome
PCOS	Polycystic Ovary Syndrome
AMH	Anti-Müllerian hormone
NIH	National Institute of Health
AE-PCOS	Androgen Excess and Polycystic Ovary Syndrome Society
BMI	Body Mass Index
HDL	High-density lipoprotein

## Appendix A

### Appendix A.1

**Table A1:** Search strategy.

Line	Search	Results	Database, Date
1	(TITLE-ABS-KEY (“prevalance” OR “prevalence”) AND TITLE-ABS-KEY (“polycystic ovary syndrome” OR “PCOS”)) AND PUBYEAR > 2017	2078	Scopus 24 April 2024
2	(“polycystic ovary syndrome”[MeSH Terms] OR “polycystic ovary syndrome”[Title/Abstract] OR “PCOS”[Title/Abstract]) and (“prevalence”[MeSH Terms] OR “prevalence”[Title/Abstract] OR “prevalance”[Title/Abstract]) and ((“2018/01/01”[Date-Publication]: “3000”[Date-Publication]))	874	PubMed 24 April 2024
3	(TI (“polycystic ovary syndrome”) OR AB (“polycystic ovary syndrome”) OR TI (“PCSO”) OR AB (“PCOS”) AND (TI (“prevalence”) OR AB (“prevalence”) OR TI (“prevalance”) OR AB (“prevalance”)) Publication Date: 20180101-20240424	186	PsycINFO 24 April 2024

## References

[1] Teede H.J., Tay C.T., Laven J.J.E., Dokras A., Moran L.J., Piltonen T.T., Costello M.F., Boivin J., Redman L.M., Boyle J.A. (2023). Recommendations from the 2023 international evidence-based guideline for the assessment and management of polycystic ovary syndrome. Eur. J. Endocrinol..

[2] Teede H.J., Khomami M.B., Morman R., Laven J.S.E., Joham A.E., Costello M.F., Patil M., Rees D.A., Berry L., Cree M.G. (2026). Polyendocrine metabolic ovarian syndrome, the new name for polycystic ovary syndrome: A multistep global consensus process. Lancet.

[3] Singh S., Pal N., Shubham S., Sarma D.K., Verma V., Marotta F., Kumar M. (2023). Polycystic ovary syndrome: Etiology, current management, and Future Therapeutics. J. Clin. Med..

[4] Rosenfield R.L., Ehrmann D.A. (2016). The Pathogenesis of Polycystic Ovary Syndrome (PCOS): The Hypothesis of PCOS as Functional Ovarian Hyperandrogenism Revisited. Endocr. Rev..

[5] Zhu T., Cui J., Goodarzi M.O. (2021). Polycystic ovary syndrome and risk of type 2 diabetes, coronary heart disease, and stroke. Diabetes.

[6] Christ J.P., Cedars M.I. (2023). Current Guidelines for Diagnosing PCOS. Diagnostics.

[7] Vale-Fernandes E., Pignatelli D., Monteiro M.P. (2025). Should anti-Müllerian hormone be a diagnosis criterion for polycystic ovary syndrome? An in-depth review of pros and cons. Eur. J. Endocrinol..

[8] Wolf W.M., Wattick R.A., Kinkade O.N., Olfert M.D. (2018). Geographical Prevalence of Polycystic Ovary Syndrome as Determined by Region and Race/Ethnicity. Int. J. Environ. Res. Public Health.

[9] Yasmin A., Roychoudhury S., Choudhury A.P., Ahmed A.B.F., Dutta S., Mottola F., Verma V., Kalita J.C., Kumar D., Sengupta P. (2022). Polycystic Ovary Syndrome: An Updated Overview Foregrounding Impacts of Ethnicities and Geographic Variations. Life.

[10] Bozdag G., Mumusoglu S., Zengin D., Karabulut E., Yildiz B.O. (2016). The prevalence and phenotypic features of polycystic ovary syndrome: A systematic review and meta-analysis. Hum. Reprod..

[11] Yu O., Christ J.P., Schulze-Rath R., Covey J., Kelley A., Grafton J., Cronkite D., Holden E., Hilpert J., Sacher F. (2023). Incidence, prevalence, and trends in polycystic ovary syndrome diagnosis: A United States population-based study from 2006 to 2019. Am. J. Obstet. Gynecol..

[12] Chiaffarino F., Cipriani S., Dalmartello M., Ricci E., Esposito G., Fedele F., La Vecchia C., Negri E., Parazzini F. (2022). Prevalence of polycystic ovary syndrome in European countries and USA: A systematic review and meta-analysis. Eur. J. Obstet. Gynecol. Reprod. Biol..

[13] Rao M., Broughton K.S., LeMieux M.J. (2020). Cross-sectional Study on the Knowledge and Prevalence of PCOS at a Multiethnic University. Prog. Prev. Med. Progress..

[14] Marchesan L.B., Ramos R.B., Spritzer P.M. (2021). Metabolic Features of Women With Polycystic Ovary Syndrome in Latin America: A Systematic Review. Front. Endocrinol..

[15] Tavares A., Rêgo Barros R.C. (2019). The Prevalence of Metabolic Syndrome in the Different Phenotypes of Polycystic Ovarian Syndrome. A prevalência da síndrome metabólica nos diferentes fenótipos da síndrome do ovário policístico. Rev. Bras. Ginecol. Obstet..

[16] Spritzer P.M., Ramos R.B., Marchesan L.B., de Oliveira M., Carmina E. (2021). Metabolic profile of women with PCOS in Brazil: A systematic review and meta-analysis. Diabetol. Metab. Syndr..

[17] Tatarchuk T., Pedachenko N., Kosei N., Malysheva I., Snizhko T., Kozub T., Zolotarevska O., Kosianenko S., Tutchenko T. (2024). Distribution and anthropometric characteristics of Rotterdam criteria-based phenotypic forms of Polycystic ovaries syndrome in Ukraine. Eur. J. Obstet. Gynecol. Reprod. Biol..

[18] Carmina E., Nasrallah M.P., Guastella E., Lobo R.A. (2019). Characterization of metabolic changes in the phenotypes of women with polycystic ovary syndrome in a large Mediterranean population from Sicily. Clin. Endocrinol..

[19] Yang R., Li Q., Zhou Z., Qian W., Zhang J., Wu Z., Jin L., Wu X., Zhang C., Zheng B. (2022). Changes in the prevalence of polycystic ovary syndrome in China over the past decade. Lancet Reg. Health West. Pac..

[20] Bigambo F.M., Wang D., Zhang Y., Mzava S.M., Dai R., Wang X. (2022). Current situation of menstruation and gynecological diseases prevalence among Chinese women: A cross-sectional study. BMC Women’s Health.

[21] Kim J.H., Jung M.H., Hong S.H., Moon N., Kang D.R. (2022). Age-Adjusted Prevalence and Characteristics of Women with Polycystic Ovarian Syndrome in Korea: A Nationwide Population-Based Study (2010–2019). Yonsei Med. J..

[22] Park Y.J., Shin H., Jeon S., Cho I., Kim Y.J. (2021). Menstrual cycle patterns and the prevalence of premenstrual syndrome and polycystic ovary syndrome in korean young adult women. Healthcare.

[23] Goh J.E., Farrukh M.J., Keshavarzi F., Yap C.S., Saleem Z., Salman M., Ramatillah D.L., Goh K.W., Ming L.C. (2022). Assessment of prevalence, knowledge of polycystic ovary syndrome and health-related practices among women in klang valley: A cross-sectional survey. Front. Endocrinol..

[24] Dashti S., Latiff L.A., Hamid H.A., Saini S.M., Abu Bakar A.S., Sabri N.A.I.B., Ismail M., Esfehani A.J. (2019). Prevalence of Polycystic Ovary Syndrome among Malaysian Female University Staff. J. Midwifery Reprod. Health.

[25] Cao N.T., Le M.T., Nguyen V.Q.H., Pilgrim J., Le V.N.S., Le D.D., Pham C.K., Aharon D., Hill M.J. (2019). Defining polycystic ovary syndrome phenotype in Vietnamese women. J. Obs. Gynaecol. Res..

[26] Ganie M.A., Rashid A., Sahu D., Nisar S., Wani I.A., Khan J. (2020). Prevalence of polycystic ovary syndrome (PCOS) among reproductive age women from Kashmir valley: A cross-sectional study. Int. J. Gynecol. Obstet..

[27] Deswal R., Nanda S., Ghalaut V.S., Roy P.S., Dang A.S. (2019). Cross-sectional study of the prevalence of polycystic ovary syndrome in rural and urban populations. Int. J. Gynecol. Obstet..

[28] Gupta M., Singh D., Toppo M., Priya A., Sethia S., Gupta P. (2018). A cross sectional study of polycystic ovarian syndrome among young women in Bhopal, Central India. Int. J. Community Med. Public Health.

[29] Varanasi L.C., Subasinghe A., Jayasinghe Y.L., Callegari E.T., Garland S.M., Gorelik A., Wark J.D. (2018). Polycystic ovarian syndrome: Prevalence and impact on the wellbeing of Australian women aged 16–29 years. Aust. N. Z. J. Obs. Gynaecol..

[30] Maredia H., Hawley N.L., Lambert-Messerlian G., Fidow U., Reupena M.S., Naseri T., McGarvey S.T. (2018). Reproductive health, obesity, and cardiometabolic risk factors among Samoan women. AM. J. Hum. Biol..

[31] Mousa M., Al-Jefout M., Alsafar H., Kirtley S., Lindgren C.M., Missmer S.A., Becker C.M., Zondervan K.T., Rahmioglu N. (2021). Prevalence of Common Gynecological Conditions in the Middle East: Systematic Review and Meta-Analysis. Front. Reprod. Health.

[32] Mirza F.G., Tahlak M.A., Hazari K., Khamis A.H., Atiomo W. (2023). Prevalence of Polycystic Ovary Syndrome amongst Females Aged between 15 and 45 Years at a Major Women’s Hospital in Dubai, United Arab Emirates. Int. J. Environ. Res. Public Health.

[33] Memon T.F., Meghji K.A., Rajar A.B., Khowaja S., Azam A., Khatoon S. (2020). Clinical, hormonal and metabolic factors associated with polycystic ovary syndrome among Pakistani women. Rawal Med. J..

[34] Alraddadi S.M., Borzangi K.A., Almuher E.M., Albaik A.F., Aljawad L.A., Shaqroon A.A., Alhazmi J. (2018). Prevalence of Polycystic Ovarian Syndrome with Associated Risk Factors and Impact on Well-Being among Women in Reproductive Aged (18–45) Years in Al-Madinah 2017. World J. Pharm. Res..

[35] Naous E., Zouein G., Asmar S., Saad E., Achkar A., Hajj G. (2023). Phenotype Prevalence and Health-Related Quality of Life of Lebanese Women With Polycystic Ovary Syndrome. Endocr. Pract..

[36] Alfanob Z.O., Ahmed M.H., Ahmed M., Badi S., Elkheir H.K. (2022). Knowledge, Prevalence and Practice of Polycystic Ovary Syndrome among Sudanese women in Khartoum State, Sudan: The need for health education. Sudan. J. Med. Sci..

[37] Elasam A.N., Ahmed M.A., Ahmed A.B.A., Sharif M.E., Abusham A., Hassan B., Adam I. (2022). The prevalence and phenotypic manifestations of polycystic ovary syndrome (PCOS) among infertile Sudanese women: A cross-sectional study. BMC Womens Health.

[38] Salari N., Nankali A., Ghanbari A., Jafarpour S., Ghasemi H., Dokaneheifard S., Mohammadi M. (2024). Global prevalence of polycystic ovary syndrome in women worldwide: A comprehensive systematic review and meta-analysis. Arch. Gynecol. Obstet..

[39] Ma Y.C., Law K.S., Wang W.S., Chang H.M. (2025). Phenotypic variations in polycystic ovary syndrome: Metabolic risks and emerging biomarkers. J. Endocrinol..

[40] Page M.J., McKenzie J.E., Bossuyt P.M., Boutron I., Hoffmann T.C., Mulrow C.D., Shamseer L., Tetzlaff J.M., Akl E.A., Brennan S.E. (2021). The PRISMA 2020 Statement: An Updated Guideline for Reporting Systematic Reviews. BMJ.

